# Systematic review of Internet of medical things for cardiovascular disease prevention among Australian first nations

**DOI:** 10.1016/j.heliyon.2023.e22420

**Published:** 2023-11-17

**Authors:** Khondker Mohammad Zobair, Luke Houghton, Dian Tjondronegoro, Louis Sanzogni, Md Zahidul Islam, Tapan Sarker, Md Jahirul Islam

**Affiliations:** a Department of Business Strategy and Innovation, Griffith Business School, Griffith University, Nathan, QLD, 4100, Australia; b Computer Science and Engineering Discipline, Khulna University, Khulna, 9208, Bangladesh; c University of Southern Queensland, Brisbane, QLD, 4300, Australia; d Griffith Criminology Institute, Griffith University, Mt Gravatt, QLD, 4122, Australia

**Keywords:** Artificial intelligence, Cardiovascular disease, Indigenous population, Internet of medical things, Internet of things, Machine learning, Telehealth, Wearable ECG sensors

## Abstract

Chronic diseases within Indigenous communities constitute the most compelling ill-health burdens and treatment inequalities, particularly in rural and remote Australia. In response to these vital issues, a systematic literature review of the adoption of wearable, Artificial Intelligence-driven, electrocardiogram sensors, in a telehealth Internet of Medical Things (IoMT) context was conducted to scale up rural Indigenous health. To this end, four preselected scientific databases were chosen for data extraction to align with the Preferred Reporting Items for Systematic Reviews and Meta-Analysis (PRISMA) technique. From the initially collected (n=4436) articles, a total of 32 articles were analysed, being synthesised from the review inclusion criteria, maintaining strict eligibility and eliminating duplicates. None of the various studies found on this innovative healthcare intervention has given a comprehensive picture of how this could be an effective method of care dedicated to rural Indigenous communities with cardiovascular diseases (CVDs). Herein, we presented the unique concepts of IoMT-driven wearable biosensors tailored for rural indigenous cardiac patients, their clinical implications, and cardiovascular disease management within the telehealth domain. This work contributes to understanding the adoption of wearable IoMT sensor-driven telehealth model, highlighting the need for real-time data from First Nations patients in rural and remote areas for CVD prevention. Pertinent implications, research impacts, limitations and future research directions are endorsed, securing long-term Wearable IoMT sensor-driven telehealth sustainability.

## 1 Introduction

Indigenous healthcare provision is in dire need of delivery reform. A recent study indicates that more than 370 million Indigenous inhabitants live worldwide with low health standards compared with benchmark populations [1]. Indigenous communities bear a heavy burden of illness, leading to lower life expectancy, severe infectious diseases, malnutrition, depression, infant and child mortality, high maternal morbidity and mortality, rising levels of cardiovascular diseases, and other chronic metabolic disease loads [2], [3]. Chronic diseases, including obesity, hypertension, CVDs, Diabetes mellitus, chronic kidney disease (CKD), and renal failure, have become significant health complications worldwide that cause millions of deaths every year [4].

CVD, an umbrella term for heart and blood vessel conditions, is a significant concern in Australian rural indigenous communities [5]. Porykali et al. [6] reveal that the indigenous populations of Australia experience higher rates of CVD, leading to increased hospitalisations and mortality compared to their non-indigenous counterparts. This pattern is not unique to Australia, as indigenous communities worldwide face elevated CVD risks [7]. While chronic diseases associated with health disparities among these communities are well documented, it remains unclear whether existing interventions sufficiently address these issues [8]. Health inequalities persist, with the indigenous population bearing a disproportionate disease burden [9]. Crengle et al. [10] demonstrate a high prevalence of clinical diagnoses related to CVD among indigenous populations in Australia and Canada [10], highlighting the substantial health disparity [9].

Telehealth/telemedicine, particularly when integrated with intelligent wearables through the Internet of Things (IoT)/ Internet of Medical Things (IoMT), offers a potential solution to bridge healthcare access gaps [11]. These techniques substantially impact modern healthcare systems by bringing value to health seekers, providing high-quality, cost-effective services, and promoting effective remote care. This study aims to explore how IoMT-driven telehealth can improve health outcomes and reduce health inequalities among rural and remote indigenous communities in Australia, especially in managing CVD through real-time patient monitoring and optimal disease management [12], [13], [14], [15].

While prior studies have investigated chronic diseases among Australian Aboriginal communities [9], [16], [7], [17], there is a gap in understanding how smart telehealth, particularly IoMT-aided CVD care, can benefit these communities comprehensively. This study presents innovative concepts of IoMT-driven wearable biosensors tailored for rural indigenous cardiac patients and their implications for cardiovascular disease management within the domain of telehealth. This study contributes by identifying adoption determinants for IoMT-driven telehealth for regional CVD care, advocating for real-time care, and shedding light on how IoMT technologies can create a novel telehealth model for remote CVD in Australia and similar settings.

This article proceeds as follows. Section 2 begins with the research rationale and conceptual model. Section 3 labels the research methods used. Section 4 outlines the data analysis and results. Section 5 focuses on a discussion of the findings. Section 6 discusses the contribution and managerial implications. Section 7 proposes the research impacts. Section 8 provides limitations and suggestions for future research directions. Section 9 concludes the article and underlines research highlights.

## 2 Research rationale

CVD is the second most significant disease burden in Australia [18]. Among the Australian First Nation people, CVD is the leading cause of disease burden and death and one of four chronic conditions that account for 70% of indigenous Australian health gaps [18]. These Australian communities suffer from heavy infectious disease loads, increasing cardiovascular and other chronic diseases, and overall poorer health indicators compared to their non-aboriginal counterparts [3]. Gibson et al. [17] remarked that chronic diseases predominantly contribute to health disparities since the life expectancy gaps peaked at 50% of Aboriginal and non-aboriginal Australians. These findings exhibit a disproportionate burden of ill health and social suffering upon Australian Aboriginal populations [9]. Unexpectedly, much less attention has been given to reducing severe health burdens, social suffering, and health gaps within this minority group and decreasing health disparity between indigenous and non-indigenous populations. Smart wearable IoMT Sensors and AI-driven telehealth would be well suited to provide cost-effective, high-quality, specialised cardiac patient care and minimise the dominating determinants of health disparity.

A plethora of contemporary research is primarily focused on the application of smart telemedicine (i.e., telehealth) for rural and remote patients using IoT technology [19], [20], [21], [22], [23], [24], [25]. These innovative IoT-aided healthcare systems are used in clinical and operational situations as part of digitally transformative practices. For example, Sawyer et al. [25] claimed that physicians make complex clinical decisions using medical big data generated by smart wearable sensors/devices. This supports [26], who asserted that big data holds great promise to create analytic models for better disease predictions, prevention, and management. Morgan et al. [23] suggested that the ground-breaking medical sensors embedded with IoMT-driven telehealth services enable patients to receive enhanced treatments and medical advice remotely. Within telemonitoring, Koya et al. [22] confirmed that an algorithm is designed to run at the gateway node to optimise the power efficiency of the sensor without causing a power drain at the gateway node. Medin-Eastwood et al. [27] stressed that wearable IoT sensors act as enablers, incessantly producing a large volume of information from structured and unstructured medical big data. This validates an underlying contribution made by these traditional technologies and Medical technologies. Broadly these are trending towards an IoT-driven healthcare ecosystem in mainstream healthcare provision.

The IoT refers to the interconnected network of physical objects (i.e., “Things”) that are integrated into the exchange of data between devices and sensors through the Internet [28]. Bajao et al. [29] revealed that IoT encompasses a network connected to the internet with various sensors, electrical chips, and relevant hardware components. The combinations of sensors technologies (MedTech) and IoT technologies have the novelty [30] to make connections between people and objects via wearables a convenient to provide them with a convenient living environment [31]. In another study, Dahlqvist et al. [30] landmark applications in diverse sectors, including smart cities, smart homes, connected cars, and e-Health/telehealth/m-health. In an application of IoT, Dwivedi et al. [28] noted that Wireless Body Area Network (WBAN) systems play significant roles in building IoT-aided Telehealth frameworks for real-time rural patient monitoring, treatment and disease management. Similarly, Riley et al. [24] illustrate how wearable biomedical IoMT sensors and AI-driven telehealth can screen patient physiological complexities and predict severe medical conditions. Dwivedi et al. [28] further pointed out that the IoMT-driven robotic technology can interact with patients after analysing medical data, providing vital signs and the status of their body to predict the risk of CVD, and recommending the necessary lifestyle changes to avoid associated complications.

**Figure 1:**
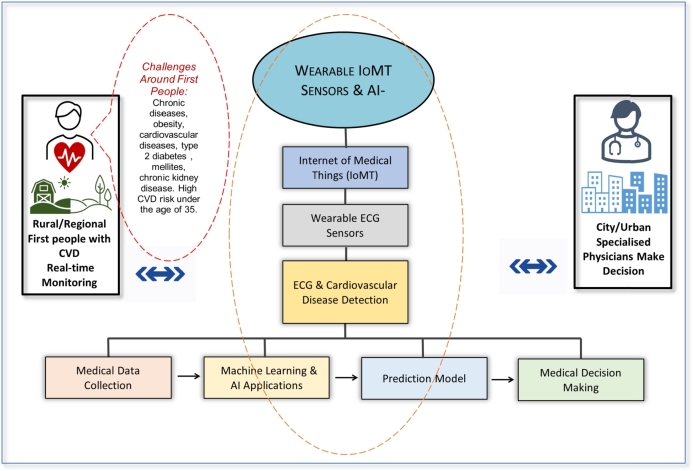
Conceptual Model of Wearable IoMT ECG AI-driven telehealth.

As mentioned above, the fast-growing innovation of machine learning (ML) and AI, further fuelled the digital transformation in healthcare services to deliver a better patient experience and optimal care. Digital health transformation technologies such as the IoMT, virtual care, real-time remote monitoring, robotic surgery, AI, Big Data analytics, smart wearables, e-health/telehealth/telemedicine, and m-health platforms are part of modern healthcare services [32]. These technologies enable the storage and sharing of relevant health information across the health ecosystems, improving medical diagnosis, the prognosis of medical risks, creating a continuum of care, and improving health outcomes, thereby creating more evidence-based knowledge for health professionals to support cutting-edge healthcare systems [32]. For instance, IoT mobile, wearable devices and smart medical sensors are instrumental in developing a smart healthcare system (i.e., telehealth). These are omnipresent, fast, and seamlessly accessible to patients [33] living in rural and remote areas. Motivated by prior research on Wearable IoMT ECG AI-driven telehealth [28], [34], [35], [25], the proposed conceptual model identifies the associations between Wearable IoMT ECG determinants and AI-driven telehealth determinants, contributing to developing a future model of care. The conceptual model in Fig. 1 investigates the research question.

This systematic review focuses on the clinical applications and evidence-based interventions of wearable IoMT ECG sensors and applications of AI-driven telehealth. In particular, this review focuses on IoT technologies that prevent the risk of cardiovascular diseases and mortality among Australian Aboriginal communities via smart telehealth ecosystems.

Meta-studies [7], [36], [17], [37] suggest research on Australian aboriginal patients with CVD has primarily been focused on conventional health care interventions. Little evidence suggests how wearable AI-driven IoMT electrocardiogram sensors can diagnose and predict Aboriginal patients' risk of CVD regardless of their geographical locations. Moreover, there is scant evidence of a robust IoMT-driven telehealth model of CVD care for these communities. These endeavours are predominantly missing and almost ignored in most existing literature in this critical field. Additionally, there has been little to no investigation into wearable IoMT ECG sensors or AI-driven telehealth models to prevent CVD risks among Australian First Nations living in rural, regional, and remote settings. The determinants of this knowledge gap ought to be filled. The present review examines the following research question:

**RQ**: *How could wearable IoMT Electrocardiogram sensors that use Artificial Intelligence-driven telehealth models be harnessed to prevent cardiovascular disease-causing morbidity and mortality among First Nations peoples living in rural, regional, and remote Australia?*

## 3 Research methodology

Systematic reviews and meta-analyses are essential tools and techniques for summarising the evidence extracted from the literature precisely, accurately and reliably [38]. This systematic review uses the standard guidelines as the Preferred Reporting Items for Systematic Reviews and Meta-Analysis(PRISMA), an approach recommended by many scholars [39], [38]. PRISMA provides comprehensive review guidelines for each checklist item related to research backgrounds, development, explanations, and rationale that reviewers can follow to ensure the undertaken review processes and meta-analysis are authentic and transparent [38].

### 3.1 Information sources

This study conducted a comprehensive peer-reviewed journal article search using four digital databases. The four digital databases, IEEE Xplore, Web of Science, ScienceDirect, and PubMed, provide a broad view of health informatics research from 2015 to 2021 and are deemed appropriate and relevant to the study's discipline. These data sources allowed us to extract a body of scholarly work on the topic, enhancing the credibility of our research findings. In addition, using four reputable databases ensured the inclusion of high-quality articles, further strengthening the validity of our systematic literature review and enabling us to provide valuable insights into the topic and contribute to the existing body of knowledge in health informatics.

### 3.2 Search approach

The search strategy for the targeted articles was limited to peer-reviewed journals from 2015 to 2021. The initial search was started on 25 July 2020 and was updated on 31 June 2021 for relevant studies that the initial search might have missed [39]. Addressing the PRISMA statement, the following search strings are used for article extraction, “Internet of Things (IoT) on cardiovascular disease prediction” OR “Indigenous Cardiovascular Disease-causing death prediction through IoT embedded Telehealth”, AND “IoT-based telemedicine”. We used a single and mix of keywords, including operators “AND” and “OR”, such as “Indigenous Cardiovascular disease and Telehealth”, OR “Indigenous-CVD-Telehealth”, “Australian Telehealth and Indigenous CVD”, “Internet of Things and Cardiovascular disease”, “Australian Rural Health and Aboriginal people”, “Indigenous people and Telehealth”, “Cardiovascular Disease and telehealth”, AND “CVD and IoT based telehealth, OR IoT and Telehealth”, “Aboriginal people and Telehealth”, OR “First people and telehealth”, “First People and Cardiovascular disease”, “Torres Islanders and Telehealth”, “Machine learning and Internet of Things based wearables”, AND “Deep learning and Cardiovascular disease monitoring using wearable IoMT sensors”, OR “Artificial Intelligence and Cardiovascular disease” AND “AI and CVD” OR “IoT and wearable ECG devices”, AND “IoT and wearable ECG sensors”, OR “Wearable CVD monitoring devices”.

### 3.3 Inclusion, exclusion and eligibility

This review included (inclusion criteria) strictly peer-reviewed published journal articles in English related to the mainstream of research; others, including duplications along with irrelevant resources, are excluded (exclusion criteria). Furthermore, this review included studies involving wearable and handheld electrocardiogram sensors, IoT technologies, as well as AI and machine learning applications. However, studies not directly related to cardiovascular disease detection, prevention, and management were excluded from consideration. It is worth noting that this review included peer-reviewed articles related to Aboriginal patients with CVDs as part of the search due to the unavailability of sufficient relevant research on wearable IoT-aided electrocardiogram sensors and AI-driven telehealth for Aboriginal patients with CVD in rural and remote Australia and globally. To ensure the appropriate article selection, the authors administered three rounds of screening and filtering processes and removed all irrelevant articles, book chapters, conference papers, and research notes, securing the most relevant items for analysis and synthesis. By adhering to stringent inclusion and exclusion criteria and employing meticulous screening and filtering processes, this review maintained a high standard of article selection, contributing to the accuracy of the study findings. The following section discusses the major themes of this research and the outcomes of the analysis. This is focused on the development of an analytic model for CVD risk prediction and policy recommendation for sustainable IoT/IoMT aided telehealth interventions for the rural and remotely living Indigenous communities.

## 4 Analysis

**Figure 2:**
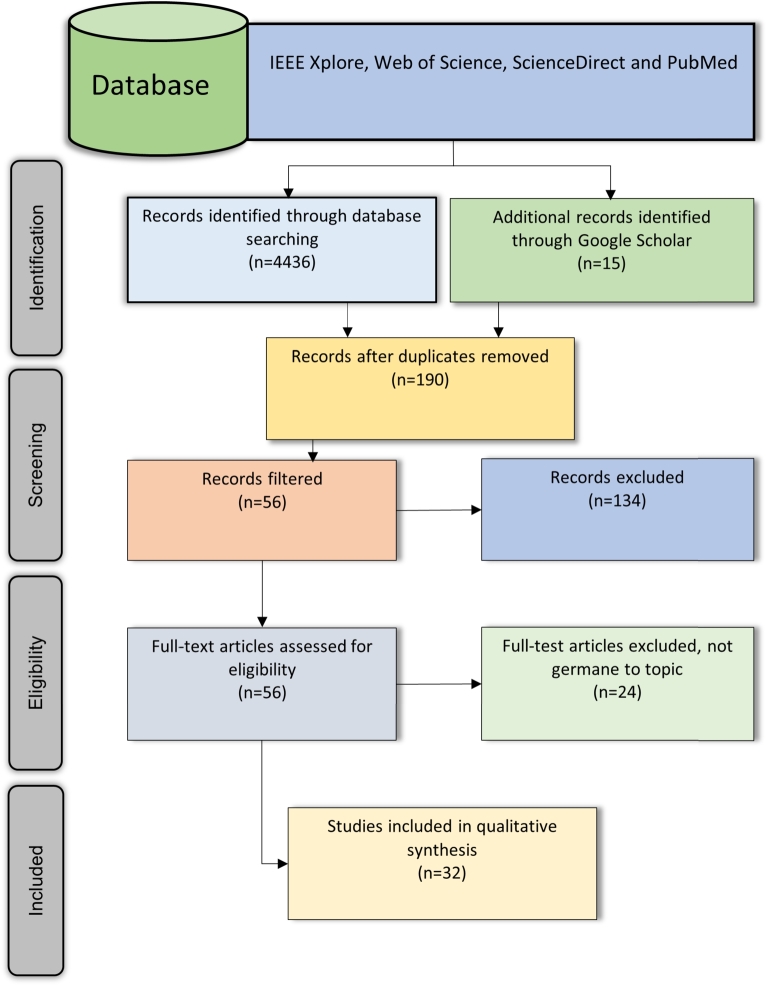
Literature search and selection process using PRISMA technique.

This study presents the review results using PRISMA guidelines (see Fig. 2) at each stage of the article classification (i.e., cleaning and filtering), adhering to the selection processes. Continuing with this approach, initially, this research yielded (n=4436) articles from four digital databases, namely IEEE Xplore, Web of Science, ScienceDirect, and PubMed. Upon the complete screening of the title, abstract, and content and duplication, a total (n=190) of papers were primarily selected that were deemed relevant to the purpose of this study. The filtering process was administered, and there were (n=56) articles included for congruency with the review inclusion criteria and (n=135) papers were excluded. Full-text articles were further assessed to meet eligibility criteria since, after careful appraisal (n=24), articles were excluded due to not being strictly pertinent to the study's subject matters. Finally, a total of (n=32) articles were finalised for data synthesis (see Fig. 2).

**Figure 3:**
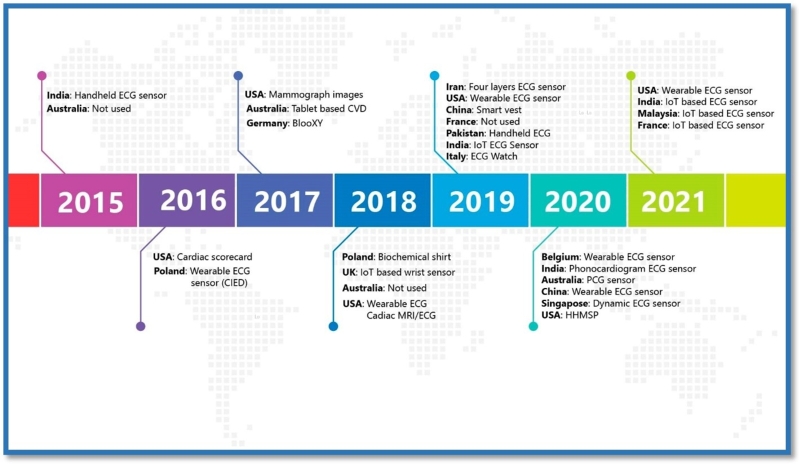
Articles published globally optimising Wearable ECG Sensors for CVD care.

**Figure 4:**
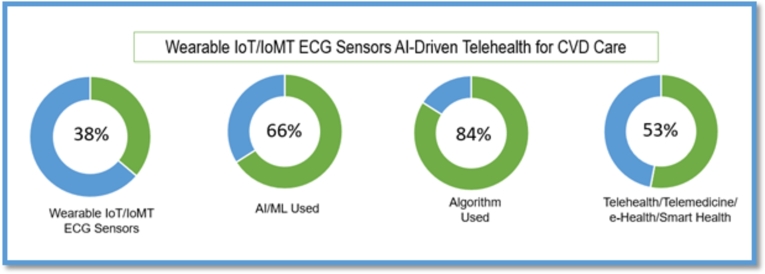
Summary Characteristics of Studies on Wearable IoT-ECG Sensors, AI-Driven Telehealth for CVDs Care.

To summarise the existing findings, Fig. 2 provides evidence of the literature search and selection process administered using the PRISMA review technique. While Fig. 1 demonstrates the conceptual model as the workflow of wearable IoMT ECG sensors, the AI-driven telehealth (IoMT, AI-TH) model for the constant real-time monitoring the patients with CVD in rural and remote areas is relevant to this study. In the present study, Table A.1 (see Appendix A) and Table B.2 (see Appendix B) describe the characteristics of the articles published within the time frame. Figure 3, Figure 4 exhibit the summary characteristics of diverse applications of wearable IoT-aided ECG sensors and AI used to diagnose and prognosis the CVD described in the literature. Moreover, our findings indicate that (19%) of studies validate the compatibility of wearable IoT/IoMT ECG sensors with AI-driven telehealth for CVD care across diverse settings. The key findings to be addressed in the following sections are the reviews.

### 4.1 Statistical and geographical distributions of published articles

**Figure 5:**
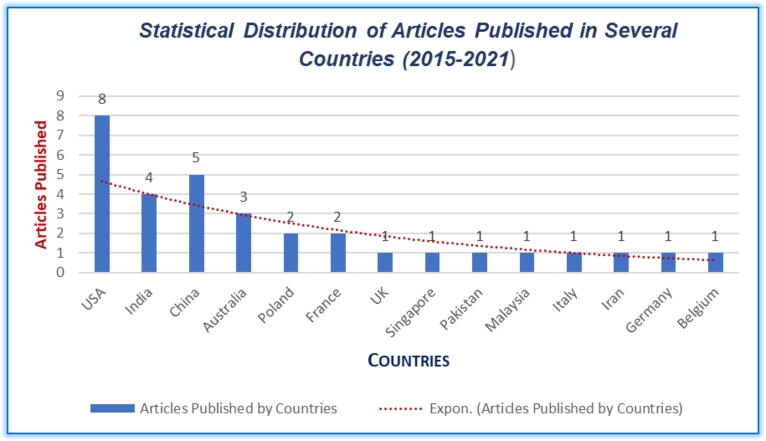
Statistical Distribution of Articles Published in Several Countries.

Fig. 5 shows the number of articles published from 2015 to 2021, indicating an overall increasing tendency, particularly in the USA (25%), accounting for eight articles, followed by China (15.62%), and India (12.5%) each contributing five and four respectively. Similarly, Australia (9.37%), France (6.25%) and Poland (6.25%) each produced 3 and 2 articles, respectively. Meanwhile, the UK (3.12%), Germany (3.12%), Belgium (3.12%), Italy (3.12%), Singapore (3.12%), Malaysia (3.12%), Iran (3.12%), and Pakistan (3.12%) each presented similar papers in the review. The inclusion of works from various countries offers a broader view of IoT/IoMT sensors-driven telehealth models for rural CVD care, ensuring a more holistic analysis and synthesis of the research landscape.

This distribution provides deep insight into the global interest and active engagement of various countries in research related to Wearable IoT-ECG Sensors, AI-Driven Telehealth for CVDs care. This validates that the USA, China, and India have been particularly active in generating new findings on this innovative model of care, while other countries also have important contributions with fewer publications.

**Figure 6:**
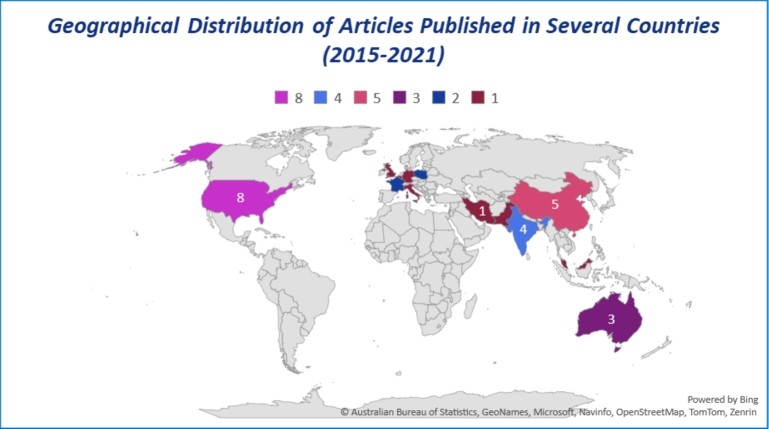
Geographical Distribution of Articles Published between 2015 to 2021.

Furthermore, the varied geographical distribution of research contributions from countries like the USA, China, and India (see Fig. 6) reflect their dominant engagements in this emerging field of research, reinforcing the innovative nature of this model of care. The variation in the number of studies from these countries suggests disparities in research emphasis, adequacy of funding and prevalence of the topic interest. This distribution also helps identify potential collaborations in research within these countries, offering significant opportunities for further exploration and cross-country comparisons. Additionally, the insights gained from this diverse pool of literature will better identify the determinants of adopting IoT/IoMT wearable ECG sensors and AI-driven telehealth models within Australia and similar settings. Finally, the findings corroborate the identification of potential gaps and opportunities for future research and practices within this innovative field.

### 4.2 Wearable IoT/IoMT sensors, AI-driven telehealth platform

IoT/IoMT-driven telehealth services have gained intensive attention and sparked interest in successful adoption to create clinical and economic values amongst diverse global health providers. The continuous evolution of technology and artifacts plays a key role in developing IoT/IoMT-based telehealth services to ensure high-quality service, safe and secured health access with affordable and reliable coordinated care for rural and remote patients. Furthermore, with the growing recognition of the roles of the IoT/IoMT and the multi-purpose biomedical devices and their interoperability of functions, there is an increased impetus for building patient-centric ‘smart’ healthcare systems. For example, Jin et al. [40] asserted that the IoMT technology has gradually been used in remote patient monitoring, screening and treatment using various innovative (i.e., MedTech) medical sensors and devices. The authors proposed a multi-dimensional predictive model based on BP neural network [40]. The proposed study incorporates multi-dimensional data analysis and achieves high prediction accuracy as an important guiding significance for intelligent medical treatment.

To determine the sustainability of IoMT-based telehealth for cardiovascular care in rural settings, Hamil et al. [21] proposed a wearable IoMT ECG, AI-driven telehealth model for arrhythmias of cardiovascular disease detection, prediction and management. The proposed wearable ECG can capture the bio-signal data and analyse them using AI and ML approaches. The authors found this model has achieved high prediction accuracy peaking at 99.56%, demonstrating the future opportunity of a large-scale deployment. Throughout this review, we will use IoT and IoMT interchangeably [21]. Utilising IoT in the MedTech industry context, Albahri et al. [19] mapped the lifecycle and architecture of wearable IoT-based telemedicine (i.e., telehealth) healthcare framework comprising IoT wearable sensors, network communications, cloud computing, hardware devices, smartphones, and AI technique. The authors in [19] further show that the IoT-based healthcare systems (LoRaWAN) contain four essential elements: data collector, analysis, treatment plan generator and treatment plan executor. Similarly, Grooby et al. [41] designed a wearable IoT phonogram (PCG), an AI-driven telehealth model for the automatic estimation of heart rate (HR) and breathing rate (BR) of CVD care. The performance and reliability of signal detection have been tested for the proposed PCG [41]. A total of 88 ten-second-long chest sound samples were taken from 76 preterm and full-term babies since this PCG successfully detected high-quality sound data and analysed them using AI and achieved a high prediction accuracy of heart rate and breath rate accounting for 93% and 82%, respectively, elucidating a robust model of CVD care for neonatal CVD patients for telehealth applications.

Taking a new approach to wearable IoT ECG sensor-based telehealth for CVD management, Koya et al. [22] distinguished the era of the IoT and hyperconnection with an ECG telemonitoring via WBAN or cloud-based within telehealth framework. The authors found these models are easy to use, self-configured, secure, plug-and-play systems with minimum hardware. Furthermore, Koya et al. [22] investigated the adaptability of smartphones as an IoT gateway for sending and receiving data to a remote server. Mobile IoT gateways offer high potential due to their widespread usage, small size with relatively high computational power, and seamless wireless connectivity [22]. Similarly, integrating more technologies into wearable IoT ECG sensor-driven telehealth systems, Beach et al. [12] proposed a wearable IoT Wrist-Worn ECG sensor to monitor home-based patients with CVDs in out-of-clinic settings in the UK. This wearable offers low-power consumption for real-time CVD patients' heart rate variability monitoring systems. These studies conclude that wearables IoT/IoMT ECG sensor has great potential for rural and remote CVD care.

Within IoMT-enabled applications, Sanamdikar et al. [15] proposed an IoMT-integrated electrocardiogram (ECG) sensor (IoMT) to monitor five different forms of beat arrhythmias, including regular, supraventricular ectopic beats, ventricular ectopic beats, the fusion of ventricular and normal, and fusion of placed and normal (N, S, V, F, Q) for early detection of heart problems of CVD patients. The findings reveal that the proposed algorithm successfully predicted cardiac arrhythmias, accounting for 98% of accuracy in feature extraction, classifications, and arrhythmia detection validating tremendous value to the patients with CVDs [15]. Additionally, the authors further clarified how the ECG beats classification technique can improve accuracy, sensitivity, specificity, and precision associated with detecting cardiac arrhythmias [15]. Dwivedi et al. [28] presented a comprehensive guideline of the IoMT structure and its competitive advantages in the healthcare system, along with various potential applications. The authors classified components of IoMT into several categories, given the full details concerning the methods and analysis used in the study.

From the prolific growth of MedTech, Rashid et al. [42] proposed a portable ECG-based telemedicine model for real-time monitoring of patients with CVD residing in non-clinical environments such as the home, office, or remote rural areas. This lightweight, portable ECG sensor enables sensing patients' heartbeat, amplitude level, and PQRST wave via the Atmega-32 microcontroller using the RS-232 serial module. Likewise, Randazzo et al. [43] designed wearable, wireless-based ECG Watch wrist-worn sensors for real-time patients with CVD monitoring. The proposed algorithm detects possible atrial fibrillation episodes within 10 seconds through a smartphone or desktop App. The authors verified that this wrist-worn ECG Watch sensor performed well in diagnosing and prognosis of atrial fibrillation disease since this cannot be easily detected in reality [43]. Interestingly, both proposed (portable ECG and wrist-worn ECG sensors) are designed for telehealth applications, but none was implanted with IoT/IoMT platforms. Likewise, Wang et al. [44] proposed a dynamic ECG compatible with telemedicine to prevent and diagnose CVD patients in Singapore. The authors applied ECG signal analysing algorithms for external noise reduction captured by ECG and found noise reduction performance outstanding. However, these sensors could have been tested with IoT/IoMT platforms for validity checks within the telehealth domain. To identify what elements of wearable IoT/IoMT-based ECG sensors are compatible with the telehealth framework for rural and remote CVD care.

### 4.3 CVD challenges in Australian aboriginal communities

Research indicates that chronic disease is the single leading cause of death among Aboriginal and Torres Strait Islander peoples in Australia [17]. Gi bson et al. [17] reviewed and highlighted the enablers and impediments related to adopting a primary healthcare model to support indigenous populations with chronic diseases in Australia, New Zealand, Canada and the USA. Their findings have some similarities to the other publications. For instance, Calabria et al. [7] found that the CVD risk is consistently higher in indigenous than non-indigenous populations indicating that Native American, Canadian First Nation, Māori, and Australian Aboriginal and Torres Strait Islander communities are at increased risk against their counterparts. Interestingly, the authors found a high absolute CVD risk in young Australian Aboriginal and Torres Strait Islanders under the age of 35 years [7], vividly highlighting health inequalities between the two groups. Unfortunately, research, strategies, policy implications, and individual and community-based efforts are not being sufficiently put forward to recognise the realities of chronic diseases. Health and well-being are also being ignored, pointing to direct and indirect health disparities between Aboriginal and non-aboriginal communities [9].

Apart from other chronic diseases, coronary heart disease (CHD), which may appear without any symptoms of cardiovascular disease, is the leading cause of morbidity and mortality worldwide [45]. The authors presented an invasive CVD diagnostic measurement and evaluation of aortic stiffness in the carotid-femoral pulse wave velocity (PWV) index to diagnose patients with CVD [45]. This index explains how the velocity of arterial pulse moving along the vessel wall indicates and predicts possible CVD events. The authors further clarified the antecedents to CVDs causing events comprising age, sex, blood pressure (BP), and heart rate (HR), all tied to be substantial auxiliary factors of aortic stiffness representing an essential index for the CVD diagnosis [45]. Moreover, they used cutting-edge AI (i.e., Artificial Neural Network) technology to explore a new CVD characteristics/elements pattern that could effectively detect coronary heart disease, prevention, and management [45].

From an early detection point of view, Shomaji et al. [46] proposed a novel wearable diagnostics system for CVD patients in the USA. These authors outlined a set of CVD diagnostic tools that can assist physicians in detecting heart diseases, including CT heart scan, chest X-rays, blood tests, cardiac catheterisation, heart MRI, stress test, pericardiocentesis, myocardial biopsies, and coronary angiography. Further, they designed the essential hardware components for wearable imaging systems and an algorithm to predict intima-media thickness (IMT), an indicator of CVDs. Likewise, in Belgium, De et al. [47] offered a multi-parameter wearable sensor for follow-up cardiac rehabilitation patients. The authors applied AI and ML approaches to visualise the relationships between sensor-derived biomarkers and sensors' capability to monitor remote CVD patient tracking. Lin et al. [35] presented an IoT-aided wearable ECG sensor for real-time patient monitoring with CVDs. They found the five different sensors that can be used to detect cardiovascular diseases such as Pulse Wave Velocity (PWV), electrocardiogram (ECG), phonocardiogram (PCG), Seismocardiogram/ballistocardiogram (SCG/BCG) and apexcardiogram (ACG). The same was found in the study by Yang et al. [48], who proposed an IoT-cloud-based ECG sensor for real-time patients with CVDs monitoring. These authors classified bio-signal data into five different ECG signal categories (P wave, T wave, Q wave, R wave and S wave) that should allow physicians to diagnose cardiovascular diseases [48].

Creating value in chronic care for Aboriginal populations via telehealth, Brazionis et al. [16] proposed a telehealth model for remote and very remote indigenous patients with CVD and Diabetes. Due to insufficient data availability, the authors could not strongly conclude whether telehealth applications can facilitate best practices in CVD and Diabetes care and disease management in remote indigenous communities in Australia. This unanswered question demands an avenue of research for this exciting new area. Table A.1 and Table B.2 (appendix) provide evidence that almost all studies used wearable ECG sensors for CVD care. At the same time, the aggregation of findings Table A.1, Table B.2 (appendix), and Figure 3, Figure 4, Figure 5, Figure 6 confirm that existing literature recognized the potential significance of IoT/IoMT-ECG sensors AI-driven telehealth for rural and remote CVD care.

### 4.4 Wearable IoT/IoMT ECG sensors-based CVD patients monitoring

In Fig. 3 and Table B.2 (appendix), diverse applications of wearable IoT-aided ECG sensors to diagnose and prognosis CVD reported in the literature were exhibited. Much work on various wearable, handheld, and dynamic ECG sensors integrated into IoT, artificial intelligence-based algorithms-driven sophisticated systems to real-time monitoring patients with CVDs proposed by Hamil et al. [21]. For instance, various ECGs are made of flexible materials, lightweight, and low-cost and are successfully and purposefully used for biomedical sensing. For example, Balsam et al. [49] proposed a biomedical shirt-based electrocardiography (ECG) sensor to monitor patients with CVD in various clinical situations. The proposed wearable ECG sensor uses Nuubo ECG systems, enables monitoring of patients with CVD, captures high-quality ECG recordings and ensures comfort for patients while wearing a biomedical shirt [49]. This novel wearable ECG technology was tested using four independent patient groups with CVD comprising patients after pulmonary veins isolation (PVI) procedure, cardiac resynchronisation therapy recipients (CRT), patients during cardiac rehabilitation after the myocardial infarction, and paediatric patients with supraventricular tachycardia (SVT) [49]. The authors found this highly effective in improving CVD diagnosis in different situations since this is washable, allowing greater patient comfort and cost-effectiveness. The biomedical shirt ECG is used in continuous real-time recordings with a battery life lasting up to 36 hours [49]. Additionally, this wearable ECG has become viable for clinical applications certified by European Union [49].

From advances in medical sensors and leveraging patients' care point of view, Lin et al. [35] summarised various sensor technologies and their flexible bio-signal sensing mechanisms, what is known as an electrocardiogram (ECG), phonocardiogram (PCG), seismocardiogram/ballistocardiogram (SCG/BCG), and apexcardiogram (ACG) used for managing cardiovascular diseases in China. Lin et al. [35] explicated how these sensors capture bio-signals, pulse wave signals, and the characteristics/elements that play key roles in CVD incidents. Similarly, Borujeni et al. [50] presented a four-layer IoT-driven intelligent healthcare system for real-time monitoring of patients with cardiovascular diseases. In the proposed model, a patient's vital signs are measured using a body sensor network and sent to an intelligent healthcare domain. Their findings confirm a significant improvement of 70% in response time and scalability compared to the state-of-the-art techniques. Another study by Al-Alusi et al. [51] configured several groups of wearable ECG sensors comprising AlivCor Kardia devices, AlivCor Apple Watch Series 4, and several others are available for clinical use since these wearables are commonly integrated into the health network infrastructure. For instance, QardioCore and Hexoskin are chest-worn-based sensors capable of recording high-quality ECG signals and tracking patients when placed in their bodies [51], thereby helping physicians manage patients with CVDs even remotely.

By optimising CVD care, advancing detection and diagnosis via ECG sensors, Baghel et al. [52] designed a phonocardiogram (PCG) for automated and real-time cardiac disease diagnostic systems that detect the present heart conditions of remote patients. Baghel et al. [52] used machine learning, AI-based (i.e., convolutional neural network) algorithms for biosignal data classifications and analysis captured by PCG sensors. They achieved high model accuracy, peaking at 98.60%, validating the robustness for multi-cardiac disease prevention, prediction and management [52]. The authors further highlighted the functional features of the PCG, which is compatible with any computing device, single-board computing processors, and Android handheld devices. Similarly, McRae et al. [53] proposed a Cardiac ScoreCard - a multivariate index assay system for early detection and frequent monitoring of traditional risk factors along with novel biomarkers for patients with CVDs. The proposed Cardiac ScoreCard system exhibited high-performance functionality and diagnostic accuracy. Another study by Pevnick et al. [54] presented broadened features of existing medical wearables adopted and accepted by many physicians for patients' heart rate and heart rhythm thoracic fluid monitoring. Behind these, to better understand characteristics and reap the benefits of various other wearables points of view, authors provided recommendations for future wearables and their potential in disease management and adoption impediments that must thoroughly be addressed [54].

With the pervasive application of wearables (i.e., ECG) from clinical practices, Sanamdikar et al. [15] implemented an IoT-based ECG that categorises five different beat arrhythmias (N, S, V, F, U), which are essential to identify a patient's heart problems. The evidence from their findings suggests that the device remained pivotal in screening, detection and prediction of cardiac arrhythmias than other approaches and achieved high predictive accuracy accounting for 98% [15]. This supports Beach et al. [12] findings, who proposed a wearable IoT Wrist-Worn ECG sensor to monitor home-based patients with CVDs in out-of-clinic settings in the UK. The authors confirmed that the proposed wearable IoT Wrist-Worn ECG sensor is significantly effective, user-friendly and lightweight 50 g, including the strap, has low power consumption, and is compatible with SPHERE (Sensor Platform for Healthcare in a Residential Environment) smart home architecture [12]. Similarly, Florez et al. [55] demonstrated an efficient wearable, IoT-aided BlooXY sensor for cardiovascular disease control, prevention, treatment, and management. The proposed IoT-based BlooXY sensor can sense and monitor patients' blood pressure, heart rate, and blood oxygen level (oximetry-SPO2), which are the essential characteristics of CVD detection and prevention [55].

### 4.5 Applications of AI and ML in CVD care

Widespread adoption interest in machine learning technology has revitalised the field of data science, and AI-powered applications have become a driving engine in many organisations [56], including health care. Tsay et al. [56] provided an overview of how AI and ML approaches promote real-time care for patients with CVDs. In this context, the authors outlined the strategies to strengthen existing clinical processes to increase accessibility, effectiveness, efficiency and availability of CVD care. Bini et al. [57] asserted that ML as a subset of AI is experiencing exponential growth in healthcare applications and has a profound impact on care delivery refinement. The author's purpose is to demystify these technology innovations for practising data scientists so they can better grasp how and where to apply them [57].

AI applications for diagnosis and prognosis are sustained in various branches of health, including oncology, dermatology, neurology, and cardiology [21]. For instance, Raj et al. [14] proposed automated handheld arrhythmias detection ECG systems for CVD diagnosis in India. The authors presented high-performance metrics that yield an overall accuracy peaked at 92.81%, 92.68% and 92.42% with average sensitivity, specificity and positive predictivity, respectively. Krittanawong et al. [58] asserted that deep learning (DL) is well-suited to cardiovascular medicine. Fig. 4 illustrates that the wearable IoT/IoMT ECG sensors AI-driven telehealth for CVD research peaked at 38% between 2015 and 2021. Likewise, ML, DL, and AI-powered diagnostic tools used for various wearable ECG sensors' sensing bio-signal data analyses for CVD detection, prediction, management and control articles peaked at 66% since different algorithms used papers peaked at 84%. In addition, wearable IoT/IoMT aided ECG, AI-driven telehealth/telemedicine/e-Health/Smart Health papers peaked at 53%. These findings (see Fig. 4) validate the continued growth of digital healthcare infrastructure along with virtual CVD care research in this exciting field.

A plethora of literature indicates that cardiovascular diseases are largely preventable but unpredictable due to underlying risk factors that may appear without any symptoms or compliance [45]. Taking this severe challenge of developing novel ML and AI-driven methods for CVD risk prediction is of immediate scientific and practical interest [45].

Similarly, Faust et al. [59] proposed a cost-effective hybrid IoT and advanced AI-based Heart Health Monitoring Service Platform (HHMSP) for CVD management. The proposed hybrid model advocates that humans and computers work together to improve cost efficiency while maintaining the reliability of diagnosis and prognosis of the CVDs [59]. From ML and AI in clinical care point of view, Bini et al. [57] presented how AI can act as a tool amplifying human cognitive functions for health providers to provide healthcare support to increasingly complicated patients. Krittanawong et al. [58] revealed that deep learning is deemed an appropriate method for cardiovascular medicine. Hemodynamic and electrophysiological indices are constantly captured by wearable sensors and image segmentation in cardiac imaging. Tsay et al. [56] demonstrated how AI platforms improve the operational delivery of cardiac care. These corroborate that AI and ML integrated into the health domain should keep pushing forward towards the novel future journey of chronic care for rural and remote communities.

From an IoT, phonography AI-driven telehealth point of view, Grooby et al. [41] offers a new approach to heart rate and breathing rate estimation from noisy neonatal chest sounds. The evidence from their proposed model demonstrated high accuracy in prediction, accounting for 93% heart sound and 82% lung sound, bolstering future telehealth applications for CVD detection, prevention, treatment, management and control in rural and remote Australia. Another study by Hamil et al. [21] designed a secured IoT, ECG AI-driven telehealth for predicting the automatic identification of arrhythmias (cardiac state) and achieved high accuracy peaking at 99.56%. The proposed model showed functional robustness, allowing a good balance between low costs and high performance while maintaining ease of use with prompt access to multiple bio-signals, thereby preventing loss of life during patients' critical situations [21].

Ma et al. [60] investigated Atrial fibrillation (AF) for CVD events. They found it is one of the most common arrhythmias related to CVDs which is difficult to monitor in real-time monitoring due to its intermittent nature. These authors proposed a wearable ECG, AI-driven telemedicine for AF detection and prevention. The proposed model achieved the highest sensitivity accounting for 99.3%, specificity of 97.4% and prediction accuracy of 98.3%, demonstrating an outperforming model of CVD care [60]. Similarly, Wang et al. [44] presented how a dynamic ECG sensor embedded with telemedicine can be implemented for real-time patient monitoring and diagnosing and preventing CVD events. The authors used a deep neural network to demonstrate how external environmental interferences (noises) could be reduced from the dynamic ECG signal classifications [44].

From an AI-powered ECG signal analysis and prediction point of view, Al-Alusi et al. [51] revealed that sensor manufacturers create algorithms that interpret ECG sensing bio-signal data governed by all the same parameters, such as negative predictive values. For example, Apple Heart Study sets a target sensitivity and specificity for their devices (Apple Watch Series 4), accounting for 92% and 90%, respectively, for AF detection algorithm [51]. A recent study by Baghel et al. [52] presented the performance and prediction accuracy of various algorithms used for analysing phonocardiogram (PCG) signal data to diagnose and prognosis of cardiac diseases. The proposed algorithms of Support Vector Machine (SVM), Random Forest (RF), Artificial Neural Network (ANN), Deep Neural Network (DNN), K-Nearest Neighbour (K-NN), Convolutional Neural Network (CNN) without augmentation, and Convolutional Neural Network (CNN) with augmentation peaked at 87.65%, 97.78%, 95%, 89.30%, 96.50%, 96.23% and 98.60% respectively leading its high accuracy and robustness to automatically diagnose and predict cardiac disorders from the PCG signals [52]. Similarly, Hamil et al. [21] presented a novel wearable IoT, ECG sensor, AI-driven telehealth model with secure wireless transmission and classification of the bio-signal platform and Xbee module with Arduino Uno and Raspberry Pi as data acquisition and processing. Authors used ECG signal data for arrhythmias (i.e., CVD event) prediction using different AI algorithms and ML methods, comprising ANN, CNN, SVM, KNN, and RF and the best classification accuracy achieved accounting for 99.56%, [21].

## 5 Discussion of the results

This is the first study to shed light on the feasibility of adopting wearable IoMT ECG sensors, an AI-driven telehealth model for rural communities, especially suitable for Aboriginal patients with CVDs living in rural and remote Australia. This review revealed that the wearable IoMT ECG sensors' AI-driven telehealth model (see Fig. 1) delivers tremendous value to rural patients in an innovative way transforming their journey towards preventive and predictive CVD care. Fig. 3 summarises IoT/IoMT embedded wearable ECG, AI/ML, algorithm, and telehealth as analysed from the reviewed studies. The aggregation of findings from the analysis (see Table A.1, Table B.2 (Appendix A and Appendix B), and Figure 3, Figure 4, Figure 5, Figure 6) concurs that the adoption of the wearable IoMT ECG sensors AI-driven telehealth continues to accelerate potential opportunities in reducing health inequalities between urban and rural counterparts. The results from the studies from 2015 to 2021 (see Fig. 3) have grown exponentially and exceeded the numbers from previous years concerning IoT/IoMT-wearable ECG sensors and AI-driven telehealth for CVD care. These findings validate the assertion that empirical research strongly emphasises the significance of this innovative model of care. This could unfold future models of CVD care for rural, regional, remote, and very remote patients with CVD regardless of the indigenous and non-indigenous communities in Australia and similar settings. Referring to health disparity between two groups, Power et al. [61] found stark health disparities between Aboriginal and non-aboriginal Australians. Another study by Haynes et al. [1] described the colonial legacies resulting in trauma, loss, and grief, contributing to a range of inequitable health and well-being outcomes. Prior research by Adelson et al. [9] asserted that health disparities point to underlying various causes of the imbalances that constantly reside outside the typically constituted health domain.

Empirical evidence from the review shows that chronic disease threat differs between the indigenous and non-indigenous populations in Australia and globally. The assumptions from the study provide evidence that CVDs have a potential impact on human health in general since the treatment period is extended, thus posing a significant threat to patients' health [35]. On this basis, a recent study by Heraganahally et al. [62] demonstrate chronic respiratory conditions among indigenous inhabitants are highly predominant, particularly in English-speaking countries. However, there appears to be significant knowledge gaps concerning indigenous inhabitants in non-English speaking countries. Haynes et al. [1] show how chronic Rheumatic heart disease predominantly impacts young people with the contemporary age-standardised occurrence at 60 times higher in the Australian aboriginal population than non-Aboriginal Australians <55 years of age. We found that demographic characteristics are dominant factors contributing to chronic disease prevalence between indigenous and non-indigenous groups. For example, Brown et al. [63] revealed that the Indigenous population's age-adjusted cardiovascular disease death remained the most significant single cause of death and was three times higher than in the non-Aboriginal community.

With regard to age factors dominantly influencing high mortality, Brown et al. [63] further illustrated that age-specific CVD causes mortality rates to even worsen between the ages of 25 and 54, peaking at 7 and 12 times that of non-Indigenous populations. Geographical factors also significantly impact chronic disease conditions among indigenous communities. For example, CVD-causing morbidity and mortality ratios also provide important insight into the cardiovascular disease burden for rural and remote aboriginal inhabitants in Australia. In covering these issues, a recent study by Gaffney et al. [64] asserted that rural and remote residents have inadequate resources to treat and prevent chronic obstructive pulmonary disease (COPD) than their urban counterparts in America. This validates that facing higher costs involving chronic conditions, which are more challenging and expensive to treat, patients are less likely to visit physicians due to additional expenses [64]. Our findings suggest that these statistics could consider a more holistic approach to adopting wearable IoMT ECG and AI-driven telehealth systems to tackle chronic disease-causing morbidly and mortality risks and reduce health inequalities in these underserved communities.

The findings from the review confirmed that tackling a growing number of patients with cardiovascular, pulmonary, and metabolic chronic diseases requires a closer look at their symptoms [65]. This corroborates that managing these diseases remains a complex clinical task because it occurs with comorbid conditions. Effective medical treatment of these chronic diseases typically requires lifestyle and food habit changes, medication regimens, and close patient monitoring [66]. Research suggests telehealth is remarkably consistent with satisfying patient care requirements in a challenging healthcare environment [25]. To this proposition, Butten et al. [67] argue that telehealth is persistently valuable and relevant to provide primary and specialist health care for disadvantaged communities who often have unfavourable health access to mainstream healthcare compared with the general population. From a practice point of view, we found that 19% of studies confirm that wearable IoT/IoMT ECG sensors are compatible with AI-driven telehealth for CVD care. The remaining 81% of studies have not been explored within the telehealth domain, indicating a significant gap in the research that our conceptual model aims to bridge.

The evidence from the review shows that the IoMT remained a fascinating digital innovation that seems poised to cross over into human biology, technology, and medical devices to treat rural and remote communities with specialised primary care via telehealth ecosystems. For instance, Albalawi et al. [20] revealed that the IoMT enables interconnecting patients, health providers, medical devices, and machines to promote evidence-based, safe, secure, and reliable patient care. This is consistent with Zhu et al. [68], who highlighted that telehealth is shown to be tied to the effectiveness of reducing risks of heart failure, diabetes, and other chronic diseases, maintaining successful distance communications between patients and physicians and increasing patients' health outcomes compared with conventional healthcare systems. This suggests wearable AI-driven telehealth provides potential health solutions for rural and remote CVD care. This is congruent with Cronin et al. [69] explanation of smart healthcare monitoring systems using cardiovascular devices (CIED: cardiac implantable electronic devices) for remotely living patients conferred a 50% relative decrease in CVD causing deaths than attending clinics follow-up. Similarly, Yang et al. [48] offers a portable ECG integrated into an IoT-based monitoring system to diagnose remotely living patients with cardiovascular diseases. From a low-cost wearable ECG sensor on the mobile devices context, Martinez et al. [70] proposed a wearable ECG sensor from e-Health (i.e., Telehealth) Biometric Sensor Platform designed by Libelium could be used for real-time CVD patients' heart rate variability monitoring.

The findings suggest that electrocardiogram (ECG), blood pressure (BP), and blood oxygen saturation level (SpO_2_) sensors integrated into IoT-driven telemedicine enable the collecting of data from remote patients with chronic diseases (i.e., CVD). Secondly, transmitting results can be close to real-time through a remote server connected with computers located in the medical centre [71]. For instance, Albalawi et al. [20] asserted that patients with chronic diseases get alerts if their health conditions deteriorate. The sensors instantly send the recorded information to the physicians via digital health networks. As the above review demonstrates, there is growing support for implementing this technology. This is consistent with our review as it outlines various dynamic electrocardiograms for real-time cardiovascular patient monitoring and demonstrates the tremendous growth of these MedTech devices in recent years. For example, Beach et al. [12] presented a wearable wristband ECG integrated into an IoT-driven model for CVD care. Similarly, Scheffler et al. [72] designed wearable wristband ECG sensors that are compatible with telemedicine and suitable for rural patients with CVD care. These validate that this wrist-worn ECG is ideal for detecting, predicting and managing CVD care, especially for the populations (i.e., patients) living in rural regional and remote areas. For example, Cugliari et al. [73] employed machine learning and AI approaches to predict the biomarkers (i.e., biological molecules found in blood, body fluids, or tissues are the sign of disease conditions) of CVD in Italy and achieved high prediction accuracy, peaked at 90%. These authors describe how myocardial infarction, acute coronary syndrome, ischemic cardiomyopathy, coronary (carotid) revascularisation, and ischemic or haemorrhagic stroke play a key role in CVD events.

In experimental research, Pevnick et al. [54] revealed that wrist-worn ECG could measure heart rates with less than 10% error compared to slandered devices under ideal circumstances. However, these devices remain largely outside usual channels [74]. More specifically, these devices detect, transmit, store and analyse data but in a database not linked to and incompatible with traditional health records resulting in useful information being unavailable for the physician unless patients volunteer it [74]. This suggests today's healthcare systems have yet to take full advantage of IoT/IoMT-enabled sensors/devices to provide extensive medical support and keep patients healthier longer [74]. However, Kindle et al. [75] argue that integrating decision support systems (CDSS) to real-time remote patient monitoring by physicians remained a formidable challenge. It is important to recognise that the advancement of ML algorithms and large databases for CDSS development provide substantial hope that a renaissance in tele-ICU care (intensive care unit) is coming soon [75]. Likewise, Liu et al. [76] proposed classification and recognition methods of encrypted ECG data based on neural networks and found satisfactory accuracy, efficiency and feasibility compared to other solutions.

From the characteristics, adaptability, and compatibility of wearable ECG sensors, this review revealed interest in whether these novel MedTech sensors can benefit patients with CVDs. For example, Al-Alusi et al. [51] asserted that the ECG sensor technology is currently being built into wearable forms capable of real-time monitoring, diagnosing and prognosis of remote patients with CVDs. Similarly, Dwivedi et al. [28] revealed that wearable medical devices with in-built sensors enable the screening of various human body infections and transfer data to monitor the real-time status of symptomatic patients. Majumder et al. [77] confirmed that an electrocardiogram (ECG) is a non- invasive approach commonly used by physicians for measuring the different forms of arrhythmia diseases (i.e., CVD events). Although many arrhythmias are uncategorised as life-threatening, such as myocardial infarction (MI), it may lead to cardiac arrest if not responded to immediately [77]. The review demonstrated various flexible MedTech sensors are currently being used in recent years. Compared with hospital devices, wearables are smaller, have lower power consumption, and can be worn comfortably [78]. These include wristbands, smart watches, glasses, body metric textiles, and more [78]. The advantages of scalability, flexibility, lightweight, and cost-effectivity, polymer films or fabrics advocate designing diverse wearable biomedical flexible sensors [79].

### 5.1 Recent advancement (2022-2023)

With recent advancements in current literature, further findings have emerged to strongly support our case. For instance, Blake et al. [80] studied Cardiac Analytics and Innovation for CVD care from an Australian perspective and highlighted the ‘siloed’ and poorly linked nature of its healthcare data. In contrast, Deniz et al. [81] found that the determinants of AI and Big Data in m-Health adoption in remote care facilities were impossible without ensuring data privacy, security, and quality assessment. IoT-driven eHealth research by Sun et al. [82] found that the high classification accuracy of interpatient ECGs is crucial in diagnosing Arrhythmia (CVD), given the pertinent risk of misclassification in eHealth settings. To resolve such problems, DL methods must be implemented to maintain high classification accuracy in AI-driven eHealth for CVD care [83].

Similarly, to improve the accuracy of IoT-driven ECG sensors, data fusion algorithms that outperform the baseline “20 Channel RR-Interval” averaging approach by ≃54% and ≃21% at signal-to-noise ratios (SNR) of 20 dB, respectively were developed [84]. In the context of AI-driven CVD care for Indigenous populations, Jeong et al. [85] pointed out that AI and ML-based predictive models could be robust solutions to CVD care rather than conventionally-used methods of care. In an experiment, Rajkumar et al. [86] echoed the superiority of IoT and DL-based methods in predicting CVD onset with an accuracy of 98.01%, boasting an error rate of 91.11% compared to other existing techniques.

### 5.2 Summary of key findings

The findings indicate that this unique model of care delivers substantial value, transforming the journey towards predictive and preventive CVD care and improving health outcomes for First Nations people. Further, adopting IoT/IoMT sensor-driven telehealth can reduce health inequalities and accelerate opportunities between urban and rural communities. The findings also emphasised the potential of wearable IoMT ECG and AI-driven telehealth systems to address chronic disease burdens in underserved communities and minimise health gaps between urban, rural, and remote regions.

The most consistent finding in this review highlighted the potential of integrating IoMT technology with telehealth ecosystems to provide evidence-based, safe, and reliable patient care for CVD. This indicates that wearable ECG and other biomedical flexible sensors offer significant advantages such as scalability, flexibility, low power consumption, and cost-effectiveness. This research suggests that wearables, IoT/IoMT electrocardiogram sensors, and AI-driven telehealth present potential opportunities for CVD diagnosis, prognosis, and management for rural and remote patients. Nevertheless, the review proposes that wearable IoMT ECG and AI-driven telehealth have the potential to transform CVD care for rural, regional, and remote aboriginal and non-aboriginal populations, providing accessible, cost-effective, and efficient smart healthcare solutions. In conclusion, this review points out that the long-term sustainability of the innovative IoT/IoMT sensor-driven telehealth care in various settings would be an enormous challenge for Australian rural healthcare infrastructure, combined with the complexity of the public healthcare administration.

In particular, the studies highlighted the disjointed nature of Australian healthcare data, the importance of accurate ECG signal classifications, and secured data privacy in facilitating the adoption of AI and big data in remote mHealth care settings. Further, AI-based predictive models emerged as robust solutions to address these issues, emphasising the superiority of wearable IoT-driven telehealth methods for CVD care dedicated towards remote Aboriginal peoples in Australia.

### 5.3 Barriers and facilitators of wearable IoT/IoMT sensors-driven telehealth for CVD care

Following the evidence from this review, for example, [19], [52], [12], [47], [21], [1], [75] we argue that the wearable IoT ECG sensors, AI-driven telehealth methods of care have had clear benefits for CVD care for rural, remote communities globally. A key question for future research to consider is: what are the barriers that prevent this life-saving technology from being implemented in rural Australia and similar settings?

This model of care has clinically been tested in various CVD conditions (adults, children, male, and female patients), and almost all types of cardiovascular disease have been screened and predicted remotely. This novel model of care provides patients and physicians with greater opportunities and flexibilities for CVD detection, early prediction, prevention, and management. This virtual model of care appears sound and could become a benchmark model to study AI-driven telehealth within the Aboriginal health domain. We believe the successful deployment of this innovative model of care will improve rural indigenous patients with CVDs and reduce health inequality among indigenous communities. Our claims are consistent with Calabria et al. [7]. CVDs are responsible for 21% of fatal diseases burden and the most prominent health disparity between aboriginal and non-aboriginal Australians [7]. Telehealth is suitable for serving broader rural and remote patients with CVD due to its technology-driven nature. Waller et al. [87] revealed that functional telehealth serving remote patients providing real-time consultation, diagnosis (e.g., echocardiogram), monitoring (e.g., EKG, glucose monitor and patients with congestive heart failure) and mentoring (e.g., another specialist observes and provide advice a remote real-time operation and virtual ICU). In a case study, Taylor et al. [88] echoed that A Children's Mercy Hospital (CHM) in Kansas City, MO, telemedicine dominantly support children in a variety of settings incorporating primary care, speciality care, pulmonary function tests, radiographs, and echocardiograms. This case study demonstrates positive public acceptance and demand for telemedicine in rural Missouri and Kansas and has led to a massive expansion, resulting in over 2000 outpatient encounters last year with a high growth rate exceeding over 40%. CHMs facilitated telemedicine now encompasses 27 paediatric specialities across four regional locations with additional expansions underway [88].

Similarly, Albahri et al. [19] commented that the wearable IoMT ECG Sensor, AI-driven telehealth, promises a vast improvement of services for remote care without incurring high medical costs. AI-based predictive aspects in the systems can assist in avoiding delays whilst timely medical treatment even before patients with CVD reach a severe condition [19]. Likewise, Zerna et al. [89] argue that multidisciplinary stroke expertise physicians are sufficiently unavailable in many rural areas, which makes delivering appropriate CVD (i.e., stroke) care in such areas a significant public health challenge. The heart of this novel method (i.e., wearable IoMT sensors, AI-driven telehealth) of care lies in its perspective on how it benefits both remote living care seekers (i.e., Aboriginal community) and urban-based care providers (i.e., physicians) and continues to serve them well.

To summarise, the contemporary literature discussed above on wearable IoMT sensors used for CVD care has had a narrow focus on clinical practices via AI-driven telehealth, particularly for rural patients. Insufficient attention has been directed towards conducting clinical trials using a large sample size. This inadequacy is reflected in the infrastructure facilities accessed and through evidence-based clinical practices. This review also exposes that the determinants of patients' acceptance, expectations, and satisfaction with this virtual care are still unidentified. What is lacking is an insight into how wearable IoMT ECG sensors collect bio-signal data and analyse them to arrive at a course of medical treatment via telehealth (medical decisions) for rural and remote communities. To point out this could drastically reduce fatalities, treatable illnesses, and related problems substantially.

From a compatibility standpoint, it is also unclear how these various MedTech technologies are compatible with traditional IoT/IoMT technologies and fit together, shaping a smart telehealth platform to create potential opportunities for rural and remote care. This indicates that research was undertaken haphazardly in recent decades. This drawback and limitations of current literature remind us that the AI-driven telehealth systems and underlying various services/facilities are not entirely recognised, even though the need is dire, and applications are readily available. Given these outcomes, we argue that health providers, policymakers, researchers, and stakeholders demand new knowledge about each aspect of this virtual care. The potential to bring health equality outcomes and reduce fatalities in treatable conditions in these communities is paramount. This new knowledge could also be shared between and across health industries strengthening service productivity, systems sustainability, and growth in Australia and globally.

## 6 Contribution and managerial implications

This study has a threefold contribution to health informatics literature. The first contribution is the investigation and identification of the adoption determinants of the wearable IoMT sensor-driven telehealth model for rural CVD care, which continues to be an under-researched area. To the authors' knowledge, this is a novel study that sheds light on the viability of adopting wearable IoMT ECG sensors within an AI-driven telehealth framework for Indigenous patients with CVD living in rural and remote Australia. This review demonstrates that there is a need for better care in rule and indigenous communities; that is, there is an inequality of access to good healthcare because of the remoteness and inaccessibility of healthcare professionals. These inadequacies are evident in the existing research both in Australia and abroad.

Second, this review demonstrates the need for adopting a wearable IoMT sensor AI-driven telehealth model for real-time care appropriate for Aboriginal and non-Aboriginal patients with CVD living in rural and remote Australia and similar settings. The adoption of this technology widens telehealth scope in developed countries' rural contexts and could drastically improve health outcomes. Thus, we believe this research further broadens the scope of AI-based health informatics research and provides helpful directions to health providers, policymakers, health authorities and stakeholders to integrate approaches to strengthen telehealth adaptability and sustainability for locational disadvantaged communities.

Third, this study makes a significant contribution to indigenous health, particularly by clarifying further how the various IoT/IoMT and sensor (MedTech) technologies could work together to build a novel telehealth model for remote CVD care in Australia and similar settings. Further, the long-term sustainability of the smart telehealth project is one of the most dominating challenges for rural healthcare infrastructure, alongside the complexity of the rural public healthcare administration. Failure to recognise these challenges could undermine their potential efficacy and years of hard work.

From a managerial perspective, the findings from the research have important implications for information systems, particularly in health informatics research. This review's findings illustrate that this care model has been clinically tested in various CVD conditions (adults, children, males, and female patients), and almost all types of CVD have been screened and predicted remotely. This novel model of care provides patients and physicians with greater opportunities and flexibilities for CVD detection, early prediction, prevention, and management. This virtual model of care appears sound and could become a benchmark model to study AI-driven telehealth within the Aboriginal health domain. This suggests that the successful deployment of this innovative model of care will improve rural indigenous patients with CVDs and reduce health inequality among indigenous communities. Given these discoveries, health providers and policymakers should design effective strategies, develop favourable policy guidelines, and implement Aboriginal CVD management plans for achieving goals.

## 7 Research impact

The present study's underlying impacts are classified into four-dimensional categories: knowledge, novelty, Australian First Nations Peoples, and developed country. This study's ability contributed to defining, distinguishing, explaining, and interpreting wearable IoT/IoMT-ECG sensors and AI-driven telehealth adoption determinants in contexts of developed countries' rural, regional, and remote settings. It provides unique insights into rural Australian First Nations' CVD treatment regarding telehealth services. This study explored the adoption determinants of the AI-driven telehealth model for CVD care. This study focused on adopting an AI-driven telehealth ecosystem for potentially applicable indigenous communities living in rural and remote Australia and similar settings. This research highlighted how this novel model of care provides patients and physicians with greater opportunities and flexibilities for CVD detection, early prediction, prevention, and disease management. This research identified wearable ECG sensors, such as a wrist-worn, dynamic watch that can detect cardiovascular signals for early diagnosis, predict patients' current CVD conditions, and manage cardiovascular diseases by early interventions.

This study introduced smart wearable IoT embedded MedTech, functional materials, network configurations, and bio-signal detection algorithms (i.e., AI and Machine Learning/Deep Learning) and their advantages. It emphasised how innovative telehealth synchronises digital and physical therapeutic modalities, reduces remote diagnostic hurdles, facilitates adaptable, comfortable, reliable, and economical healthcare interventions, and bridges inequality gaps between urban and rural health landscapes. Further, this research identified four areas of impact, namely (1) research-related (i.e., research problem, methods used, research management and communication), (2) policy (i.e., level of policy making, type, nature and policy networks), (3) service (i.e., health services, service management, quality of care and information systems) [90].

## 8 Limitations and future research

Nevertheless, several limitations should be considered when interpreting these research findings. This review used only four scientific databases for data extraction and synthesis. The future review should include additional databases to extract more data that may influence the broad view of the phenomenon. The future search should broaden by including other chronic disease risks and management using these technologies to observe the viability and effectiveness of this model of care on a large-scale adoption. Finally, our study did not undertake a formal quality assessment of the incorporated literature, thus constraining our ability to critically evaluate the sources utilised to substantiate our assertions.

## 9 Conclusion

One relatively unexplored research area involves the broad adoption of wearables IoMT sensors AI-driven telehealth focusing on rural and remote communities. Although wearables Internet of Medical Things electrocardiogram sensors, Artificial Intelligence-driven telehealth hold increased opportunities for cardiovascular disease diagnosis, prognosis, and management for rural and remote patients. Incorporating a large volume of research on the issues discussed in this review serves as a comprehensive guideline and list of the sources to lead the way in adopting this novel model of care for the Aboriginal communities living in rural and remote Australia.

The novelty of this study has advanced a pragmatic understanding of sustaining this cutting-edge model of care tackling high risks of CVD, causing deaths, challenges, and potential future directions of continuous monitoring and ubiquitous medical treatment via telehealth for underprivileged and vulnerable aboriginal communities. The study findings highlight the essential tools and resources that should be taken into consideration by the relevant authorities for extensive adoption. The government, health authorities, policymakers, health providers, and stakeholders are urged to work together, emphasising the implementation strategies for an initial pilot project as setting up a foundation before its widespread adoption in rural Australia and similar settings. The successful adoption of a wearable IoMT sensor-driven telehealth model of care could help reduce health inequalities affecting underprivileged rural/remote minorities. Rural regional and remote populations often have limited access to dedicated public medical facilities, and most lack private after-hours medical practitioners [34]. Hence, these locational disadvantages and sparsely populated regions should continue to be a priority [34].

## Funding

The authors declare no funding for this research.

## CRediT authorship contribution statement

**Khondker Mohammad Zobair:** Writing – review & editing, Writing – original draft, Methodology, Investigation, Formal analysis, Data curation, Conceptualization. **Luke Houghton:** Writing – review & editing, Writing – original draft, Methodology, Investigation, Formal analysis, Data curation, Conceptualization. **Dian Tjondronegoro:** Writing – review & editing, Writing – original draft, Methodology, Investigation, Formal analysis, Data curation, Conceptualization. **Louis Sanzogni:** Writing – review & editing, Writing – original draft, Methodology, Investigation, Formal analysis, Data curation, Conceptualization. **Md Zahidul Islam:** Writing – review & editing, Writing – original draft, Methodology, Investigation, Formal analysis, Data curation, Conceptualization. **Tapan Sarker:** Writing – review & editing, Supervision, Project administration. **Md Jahirul Islam:** Writing – review & editing, Writing – original draft, Methodology, Investigation, Formal analysis, Data curation, Conceptualization.

## Declaration of Competing Interest

The authors declare that they have no known competing financial interests or personal relationships that could have appeared to influence the work reported in this paper.

## Figures and Tables

**Table A.1:** Literature Search results on Wearable IoT/IoMT, ECG sensors, AI-driven Telehealth for CVD care.

ID	Year	Author	Country of Studies	Study Proposal	Methods	Accuracy (%)	Objectives and Outcome Measures
1	2018	[49]	Poland	Study design and rationale for biomedical shirt-based electrocardiography monitoring in relevant clinical situations: ECG-shirt study.	Arrhythmia detecting algorithm: the Shapiro-Wilk test with SAS® software version 9.4.	98%	This study shows the utility of biomedical shirt-based electrocardiography (ECG) monitoring of patients with CVD in different clinical situations employing the Nuubo ECG (nECG) systems. The proposed biomedical shirt captures the electrocardiographic signal via textile electrodes integrated into a garment.
2	2019	[50]	Iran	A hierarchical, scalable and real-time monitoring system for electrocardiography using context-aware computing.	Wireless body area networks, Global Positioning System, Blood Pressure, survey systems, activity monitors, body temperature, and ECG signal detector.	70%.	In the proposed model, a patient's vital signs are measured using a body sensor network and sent to an intelligent health care domain. Empirical findings confirm a significant improvement of 70% in response time and scalability compared to the state-of-the-art techniques. This improvement is about 30% compared to Fog computing approaches.

3	2019	[51]	USA	Wearing Your Heart on Your Sleeve: the Future of Cardiac Rhythm Monitoring.	Arterial Fibrillation (AF) detection algorithms.	92% specificity, 90% sensitivity	This study configured several groups of wearable ECG sensors comprising of AlivCor Kardia devices, Apple Watch Series 4, and several others.

4	2020	[52]	India	Automatic diagnosis of multiple cardiac diseases from PCG signals using Convolutional Neural Network (CNN).	CNN	98.60%	This study designed a phonocardiogram for automated and real-time cardiac disease. The authors used CNN for biosignal data classifications captured by PCG sensors. The authors also highlighted that PCG is compatible with any computing device, single board computing processors, android handheld devices.

5	2016	[53]	USA	Cardiac ScoreCard: A diagnostic multivariate index assay system for predicting a spectrum of cardiovascular disease.	Lasso Logistic regression.	AUC=0.8403 for disease vs. non-case and 0.9412 for cardiac wellness vs. HF.	Cardiac ScoreCard is a multivariate index assay system for early detection and frequent monitoring of traditional risk factors along with novel biomarkers for patients with CVDs.

6	2017	[16]	Australia	Evaluation of telehealth facilitation of Diabetes and cardiovascular care in Indigenous communities.	Analysis of covariance (ANCOVA).	95%	Creating value in chronic care for Aboriginal populations via telehealth, the authors surveyed proposing a telehealth model for remote and very remote indigenous patients with cardiovascular disease and Diabetes.

7	2021	[15]	India	Classification f cardiac arrhythmia based on incremental support vector regression on IoT platform.	Incremental Support Vector Regression.	98%	This study proposed IoT integrated electrocardiogram sensor to monitor different forms of beat arrhythmias for identifying heart problems. The proposed algorithm successfully predicted cardiac arrhythmias with 98% accuracy.

8	2018	[12]	UK	An Ultra-Low Power Personalised Wrist Worn ECG Monitor Integrated With IoT Infrastructure.	Flexible textile electrodes into smart clothing.	10% variance of the mean.	Proposed a wearable IoT Wrist-Worn ECG sensor to monitor home-based patients with CVDs in the UK. This wearable offers low-power consumptions for real-time heart rate variability monitoring.

9	2019	[91]	China	Signal Quality Assessment and Lightweight QRS Detection for Wearable ECG SmartVest.	Signal Quality Assessment and QRS detection algorithms	F1 score >99.5%	Proposed an IoT-based wearable 12-lead ECG SmartVest system for the early CVD detection. The proposed method can efficiently deal with the trade-off between accepting good and rejecting poor quality ECGs.
10	2016	[13]	Poland	Telerehabilitation in heart failure patients.	Unspecified.	94.7%	Discusses the characteristics, applicability, and effectiveness of telemonitoring tools to provide optimal long-term management for heart failure patients unable to attend traditional rehabilitation programs.

11	2015	[14]	India	Development of Handheld Cardiac Event Monitoring System.	Principal Component Analysis and Feed-forward Neural Networks.	Sensitivity 92.81%, specificity 92.68% and precision 92.42%.	Proposed automated handheld arrhythmias detection ECG systems for CVD diagnosis in India.

12	2016	[48]	China	An IoT-cloud Based Wearable ECG Monitoring System for Smart Healthcare.	Unspecified.	R wave 0.68 indicates health heart.	Proposed an IoT-cloud-based ECG sensor for real-time patients with CVDs monitoring. The proposed system is reliable in collecting and displaying real-time ECG signal data, which may contribute to the diagnosis of certain patients with CVDs.

13	2020	[47]	Belgium	Wearable monitoring and Interpretable ML to track progression in patients during cardiac rehabilitation.	Support Vector Machine (SVM).	66%.	Proposed a multi-parameter wearable sensor for follow-up cardiac rehabilitation patients in Belgium. Applied a interpretable ML approach to visualise the relationships between sensor-derived biomarkers and their ability to monitor remote patients.

14	2019	[46]	USA	Early detection of CVD using wearable ultrasound device.	Wearable imaging system algorithm.	Unspecified.	Proposed a wearable ultrasound imaging device for the early detection of CVDs through a quantitative carotid artery monitoring system in the USA. The distinctive features of the device include portability, accuracy, ease of use, and cost-effectiveness. The authors outlined a set of diagnostic tools including CT heart scans, chest X-rays, blood tests, cardiac catheterisation, heart MRI, stress test, pericardiocentesis, myocardial biopsies, and coronary angiography.

15	2021	[21]	France	Design of a secured telehealth system based on multiple biosignals diagnosis and classification.	Artificial Neural Network (ANN), Convolutional Neural Network (CNN), Support Vector Machine (SVM), K-Nearest Neighbour (KNN) and Random Forest (RF).	99.56%.	Proposed a wearable IoMT ECG, AI-driven telehealth model for arrhythmias of CVD detection, prediction, and management. The proposed wearable ECG can capture the biosignal data and analyse them using AI and ML approaches.

16	2020	[41]	Australia	Neonatal heart & lung sound quality assessment for heart and breathing rate estimation.	Support Vector Machine (SVM), K-Nearest Neighbours (KNN), Decision Tree (Tree).	93% for heart sounds, and 82% for lung sounds.	Designed a wearable IoT phonogram (PCG), an AI-driven telehealth model for the automatic estimation of heart rate and breathing rate of CVD patients. Analysed a total of 88 10-second-long chest sound samples from 76 preterm and full-term babies.
17	2020	[44]	Singapore	ECG signal denoising based on deep factor analysis.	Deep factor analysis for noise reduction.	Unspecified	Proposed a dynamic ECG compatible with telemedicine to prevent and diagnose CVD patients. The authors applied ECG signal analysing algorithms for external noise reduction captured by ECG.

18	2019	[45]	France	Coronary heart disease diagnosis by artificial neural networks including aortic pulse wave velocity index and clinical parameters.	Artificial neural networks (ANNs).	63-93%	Presented an invasive CVD diagnostic measurement and evaluation of aortic stiffness in the carotid-femoral pulse wave velocity (PWV) index to diagnose patients with CVDs. The index explains how the velocity of arterial pulse moving along the vessel wall indicates and predicts the possible CVD events. Used cutting-edge AI (i.e., ANN) technology to explore a new CVD characteristics/elements pattern.

19	2018	[7]	Australia	Absolute CVD risk and lipid-lowering therapy among Aboriginal and Torres Strait Islander Australians.	Risk assessment and risk management algorithm.	Unspecified.	Found that the CVD risk is consistently higher in indigenous than non-indigenous populations including Native American, Canadian First Nation, Māori, Australian Aboriginal, and Torres Strait Islander communities.

20	2019	[42]	Pakistan	Design and implementation of real-time electrocardiogram monitoring system for telemedicine services.	MultisimTM	Unspecified.	Proposed a portable ECG-based telemedicine model for real-time monitoring of patients with CVD residing in non-clinical environments. The lightweight, portable ECG sensor enables sensing patients' heartbeat, amplitude level, and PQRST wave via a microcontroller

21	2017	[55]	Germany	BlooXY: a non-invasive blood monitor.	Used secured 128 bits asymmetric encryption for data security.	Unspecified.	Demonstrated an efficient wearable sensor called BlooXY for CVD control, prevention, treatment, and management. BlooXY is able to sense and monitor blood pressure, heart rate, and blood oxygen level.

22	2019	[22]	India	Plug and play self-configurable IoT gateway node for telemonitoring of ECG.	A self-configuration algorithm is designed to run at the gateway node to optimize the power efficiency.	Unspecified.	Assessed hyper connection with an ECG telemonitoring via WBAN. Investigated the adaptability of smartphones as an IoT gateway for sending and receiving data to a remote server.

23	2018	[56]	USA	Evaluation of AI and ML applications in real-world Cardiac Care.	Overview	Overview	Provided an overview of how AI platforms promote the operational delivery of care for patients with CVDs. Outlined strategies for integrating ML to augment existing clinical care processes to improve CVD care.

24	2018	[57]	USA	To understand the impact of AI, ML, deep learning and cognitive computing on healthcare.	Artificial neural networks and deep learning.	Unspecified.	Provide a historical perspective of AI and ML approaches and evolution of deep learning algorithms through biomimicry (biological entities and processes). Main aim is to demystify these technology innovations for surgeons.
25	2020	[60]	China	Automated classification of atrial fibrillation.	Artificial neural network (ANN).	98.3%	Proposed a wearable ECG, AI-driven telemedicine for AF detection and achieved 99.3% sensitivity, 97.4% specificity and 98.3% accuracy outperforming previous models.

26	2019	[58]	USA	Deep learning for cardiovascular medicine: a practical primer.	Deep learning.	Unspecified	Revealed that deep learning based cardiac image analysis can be useful for CVD patients care.

27	2018	[54]	USA	To provide a framework for the future wearable technologies for cardiology	Overview	Overview	Presented broadened features of existing medical wearables for patients' heart rate and heart rhythm thoracic fluid monitoring. Authors provided recommendations for future wearables and their potential in disease management.

28	2016	[48]	China	An IoT-cloud based wearable ECG monitoring system.	ECG monitoring system.	Unspecified.	Proposed a wearable IoT-driven ECG for CVD monitoring systems. ECG data are gathered using a wearable monitoring node and are transmitted directly to the IoT cloud using WI-Fi. HTTP and MQTT protocols are employed to provide visual and timely ECG data.

29	2021	[35]	China	Wearable sensors and devices for real-time CVD monitoring.	Overview	Overview	Reviews the latest developments in surveillance technologies for CVD. A variety of signals that can monitor CVD are summarised. The different mechanisms and principles of monitoring pulse signals are discussed.

30	2019	[43]	Italy	ECG WATCH: a real-time wireless wearable ECG.	Atrial fibrillation detection algorithm.	90.5%	Designed wearable, wireless-based ECG watch (writs-worn) for the real-time monitoring. The proposed algorithm detects possible atrial fibrillation episodes within 10 seconds through a smartphone or desktop App.

**Table B.2:** Characteristics of the Selected Studies.

ID	Year	Authors	Country	Disease Type	Devices/Sensors used	IoT/IoMT (Y/N)	Sample Size	ML/AI (Y/N)	Algorithm (Y/N)	Telehealth/Telemedicine/e-health/Smart health
1	2018	[49]	Poland	CVD	Biochemical shirt-based ECG	N	120	N	Y	Y
2	2019	[50]	Iran	CVD	Four layers ECG	Y	0	N	Y	Y
3	2019	[51]	USA	CVD	Wearable ECG	N	0	N	N	Y
4	2020	[52]	India	CVD	Phonocardiogram signal ECG	N	3000	Y	Y	Y
5	2016	[53]	USA	CVD	The Cardiac Scorecard systems	N	579	Y	Y	N
6	2017	[16]	Australia	CVD	Mobile Tablet based CVD management tool	N	576	N	Y	Y
7	2021	[15]	India	CVD	IoT based ECG	Y	400	Y	Y	Y
8	2018	[12]	UK	CVD	IoT based wrist-worn ECG sensor	Y	5	Y	Y	Y
9	2019	[91]	China	CVD	IoT based ECG Smart Vest system	Y	317	Y	Y	Y
10	2016	[13]	Poland	CVD	Cardiovascular implantable electronic devices (CIED)	N	0	N	N	Y
11	2021	[19]	Malaysia	CVD	IoT sensors	Y	0	N	N	Y
12	2020	[14]	India	CVD	Phonocardiogram signal ECG	N	48	Y	Y	Y
13	2016	[48]	China	CVD	IoT aided ECG sensor	N	0	N	Y	N
14	2020	[47]	Belgium	CVD	Dynamic ECG Sensor	N	129	Y	Y	Y
15	2019	[46]	USA	CVD	Wearable point-of-care imaging system	N	12	N	Y	N
16	2017	[21]	USA	CVD	Breast arterial calcifications Mammogram images	N	840	Y	Y	N
17	2021	[41]	France	CVD	IoT based ECG	N	473	Y	Y	Y
18	2020	[44]	Singapore	CVD	ECG Sensor	N	47	Y	Y	Y
19	2020	[45]	France	CVD	Dynamic ECG Sensor	N	437	Y	Y	N
20	2018	[7]	Australia	CVD	Not used	N	14350	N	N	N
21	2015	[17]	Australia	CVD	Not used	N	0	N	N	N
22	2019	[42]	Pakistan	CVD	Atmega-32 micro controller based Handheld based ECG	N	0	N	Y	Y
23	2017	[55]	Germany	CVD	IoT based Writs-worn ECG (BlooXY)	Y	37	N	Y	N
24	2019	[22]	India	CVD	IoT based ECG Sensor	Y	549	Y	Y	Y
25	2019	[56]	USA	CVD	IoT based ECG (wrist-worn)	Y	0	Y	Y	N
26	2018	[57]	USA	CVD	Wearable ECG	N	0	N	Y	N
27	2020	[60]	China	CVD	Wearable ECG	Y	0	Y	Y	N
28	2020	[58]	USA	CVD	IoT based HHMSP systems (ECG)	Y	0	Y	Y	N
29	2018	[54]	USA	CVD	ECG sensor	N	0	Y	Y	N
30	2021	[35]	China	CVD	IoT based ECG, PCG SCG/BCG/ACG sensors	Y	0	Y	Y	Y
31	2016	[48]	China	CVD	Wearable ECG Sensors	Y	0	Y	Y	Y
32	2019	[43]	Italy	CVD	IoT ECG Watch	Y	40	Y	Y	Y

**Note**: CVD=cardiovascular diseases; ECG=Electrocardiogram; PCG=Phonocardiogram; IoT=Internet of Things; IoMT=Internet of Medical Things; ML=Machine Learning; AI=Artificial Intelligence.

## Data Availability

The authors confirm that no data were used in the research described in this article. All analyses, findings, and conclusions presented in this paper are based on the published literature and theoretical frameworks. No data sets, codes, or other materials are associated with this study.
